# Organotypic human skin culture models constructed with senescent fibroblasts show hallmarks of skin aging

**DOI:** 10.1038/s41514-020-0042-x

**Published:** 2020-03-06

**Authors:** Regina Weinmüllner, Barbara Zbiral, Adnan Becirovic, Elena Maria Stelzer, Fabian Nagelreiter, Markus Schosserer, Ingo Lämmermann, Lisa Liendl, Magdalena Lang, Lucia Terlecki-Zaniewicz, Orestis Andriotis, Michael Mildner, Bahar Golabi, Petra Waidhofer-Söllner, Karl Schedle, Gerhard Emsenhuber, Philipp J. Thurner, Erwin Tschachler, Florian Gruber, Johannes Grillari

**Affiliations:** 1 Christian Doppler Laboratory for Biotechnology of Skin Aging, Vienna, Austria; 2 Department of Biotechnology, BOKU - University of Natural Resources and Life Sciences, Vienna, Austria; 3 Austrian Cluster for Tissue Regeneration, Vienna, Austria; 4 Institute of Lightweight Design and Structural Biomechanics, TU Wien, Getreidemarkt 9/317, 1060 Vienna, Austria; 5 Division for Biology and Pathobiology of the Skin, Department of Dermatology, Medical University of Vienna, Vienna, Austria; 6 Institute of Immunology, Center of Pathophysiology, Infectiology and Immunology, Medical University of Vienna, Vienna, Austria; 7 Institute of Animal Nutrition, Livestock Products and Nutrition Physiology (TTE), Department of Agrobiotechnology, BOKU - University of Natural Resources and Life Sciences, Vienna, Austria; 8 Institute of Wood Technology and Renewable Materials, Department of Material Sciences and Process Engineering, BOKU - University of Natural Resources and Life Sciences, Vienna, Austria; 9 Ludwig Boltzmann Institute for Experimental and Clinical Traumatology, Vienna, Austria

**Keywords:** Senescence, Ageing, Senescence

## Abstract

Skin aging is driven by intrinsic and extrinsic factors impacting on skin functionality with progressive age. One factor of this multifaceted process is cellular senescence, as it has recently been identified to contribute to a declining tissue functionality in old age. In the skin, senescent cells have been found to markedly accumulate with age, and thus might impact directly on skin characteristics. Especially the switch from young, extracellular matrix-building fibroblasts to a senescence-associated secretory phenotype (SASP) could alter the microenvironment in the skin drastically and therefore promote skin aging. In order to study the influence of senescence in human skin, 3D organotypic cultures are a well-suited model system. However, only few “aged” skin- equivalent (SE) models are available, requiring complex and long-term experimental setups. Here, we adapted a previously published full-thickness SE model by seeding increasing ratios of stress-induced premature senescent versus normal fibroblasts into the collagen matrix, terming these SE “senoskin”. Immunohistochemistry stainings revealed a shift in the balance between proliferation (Ki67) and differentiation (Keratin 10 and Filaggrin) of keratinocytes within our senoskin equivalents, as well as partial impairment of skin barrier function and changed surface properties. Monitoring of cytokine levels of known SASP factors confirmedly showed an upregulation in 2D cultures of senescent cells and at the time of seeding into the skin equivalent. Surprisingly, we find a blunted response of cytokines in the senoskin equivalent over time during 3D differentiation.

## Introduction

The human skin is exposed daily to internal and external stressors, which ultimately leads to an “aged“ phenotype with wrinkling, sagging, thinning of the epidermis, dry skin, and reduced barrier integrity. Currently, the complex network of intercellular crosstalk between the different cell types in the human skin is not fully understood, and it remains unclear how aged cells can have detrimental effects on tissue function. These effects cannot be easily studied in a classic, two-dimensional system, but need a more holistic, three-dimensional approach. Organotypic skin equivalents can help to close this research gap by providing a model system that allows coculture of different skin cells in a three-dimensional matrix.

## Results and discussion

Skin aging is a multifaceted process, driven by a wide range of intrinsic and extrinsic factors simultaneously affecting skin cells, ultimately leading to a progressive decline of tissue functionality. Cellular senescence is one of these factors^1^, and an estimated 20–50% of cells are senescent in the skin of elderly^2,3^. In order to study the influence of senescence in human skin, 3D organotypic cultures are a well-suited model system. However, only few “aged” skin-equivalent (SE) models are available, requiring complex and long-term experimental setups^4–6^. Here, we adapted a previously published full-thickness SE model^7^ by seeding increasing ratios of stress-induced premature senescent (SIPS) versus normal fibroblasts into the collagen matrix, terming these SEs “senoskin”.

### Senescent fibroblasts survive in a 3D environment

**Fig. 1:**
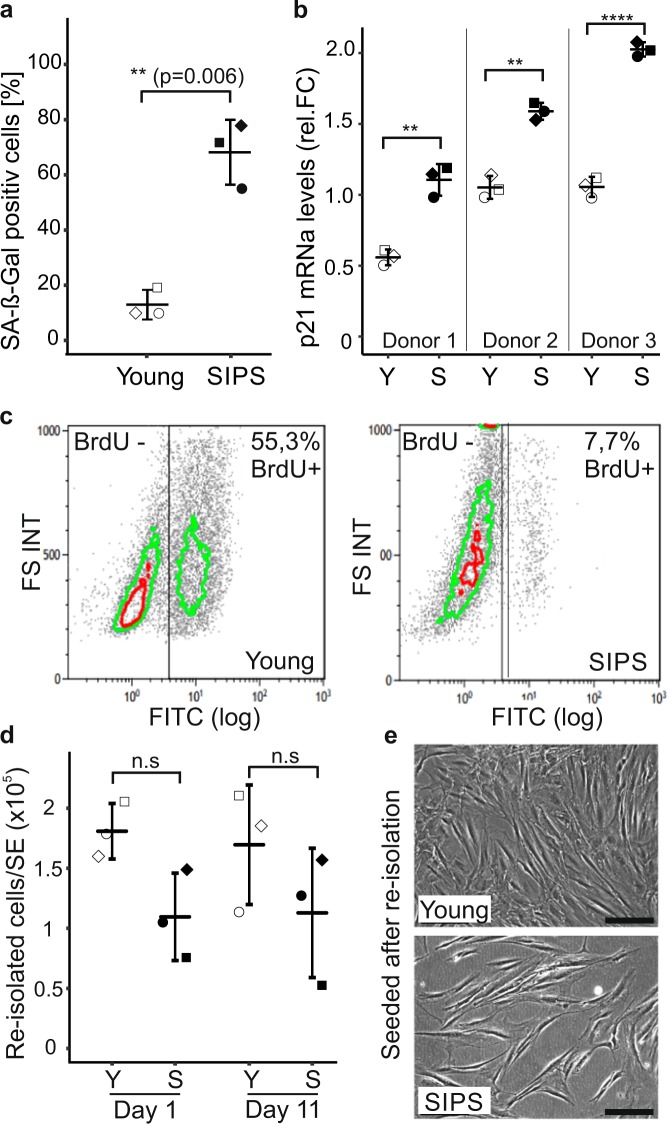
Senescent fibroblasts survive in a 3D environment. **a** Quantification of senescence-associated beta-galactosidase (SA-β-Gal). Human dermal fibroblasts (HDF) were seeded at a density of 3500 cells/cm^2^ and stressed twice for 5 consecutive days with 100 µM H_2_O_2_ each (SIPS). Cells were stained for SA*-β*-Gal after recovery for 3 days. The percentage of SA beta-gal-positive cells was evaluated by counting images of >100 cells per condition; proliferating cells (Young) served as a control. Three biological replicates are shown; error bars indicate SD. Statistical analysis was performed using unpaired *t* test. n.s ≥ 0.05; *p < 0.05; **p < 0.01. Scale bar = 100 µm. **b** Quantification of p21 mRNA levels via qPCR. Senescent human fibroblasts were harvested, and p21 mRNA levels were analyzed by real-time PCR. Quiescent cells served as a control. GAPDH was used as housekeeping gene to normalize values. Relative foldchange was calculated by setting the mean p21 value of quiescent cells to 1. Three independent biological replicates from three different donors are shown; error bars indicate SD. Statistical analysis was performed using unpaired *t* test. n.s ≥ 0.05; *p < 0.05; **p < 0.01. **c** Induction of growth arrest by SIPS treatment. Cells were incubated for 24 h with medium containing 10 µM BrdU followed by immunolabeling with murine anti-BrdU, a FITC-labeled secondary antibody, and subsequent flow cytometric measurement. Data from one representative experiment are shown. The experiment was repeated three times each in three different donors with similar results. **d**, **e** Survival of senescent cells in skin equivalents (SEs). SEs were prepared with 100% young (Young) or 100% senescent cells (SIPS) according to the protocol. The dermal part was harvested on days 1 and 11 of the experiment, and the collagen matrix was subsequently dissolved by incubation with collagenase for 1 h at 37 °C. **d** The re-isolated cells were counted manually, and the total amount of cells per skin equivalent was calculated. **e** For re-seeding, an aliquot of the re-isolated sample was transferred into a six-well plate and incubated in fresh media for 3 days at 37 °C, 7% CO_2_ before pictures were taken. Three independent replicates are shown; error bars indicate SD. Statistical analysis was performed using unpaired *t* test. n.s ≥ 0.05; *p < 0.05; **p < 0.01. Scale bar = 100 µm.

SIPS was induced by chronic sublethal exposure to H_2_O_2_^8,9^ in fibroblast strains from three different donors in three independent experiments each, and confirmed by an increase in senescence-associated beta-galactosidase-positive cells (68% vs 13%, Fig. 1a, and Supplementary Fig. 1), senescent cell-like morphology (Supplementary Fig. 1), as well as p21 mRNA levels (Fig. 1b) and y-H2AX foci in cell nuclei (4.5 foci/nucleus vs 0.37, Supplementary Fig. 1). In addition, BrdU-positive cells were decreased markedly indicating growth arrest (7.7% vs 55.3%, Fig. 1c). To demonstrate the survival of senescent fibroblasts within the collagen matrix of senoskin models, we re-isolated and counted cells at different time points. No significant decrease in senescent cell count after re-isolation was observed (Fig. 1d), indicating stable cell numbers throughout the experiment. Moreover, re-isolated cells could be seeded into new culture dishes, and assumed their original unaltered early passage or senescent cell morphology after attachment (Fig. 1e). However, we observed a trend toward less senescent cells that could be re-isolated from our senoskin model (110.000 cells/senoskin vs 175.000 cells/control, Fig. 1d). To ensure that this had no impact on skin-equivalent formation, we built skin models with half the original amount of young fibroblasts, and measured the resulting epidermal thickness. No significant decrease in epidermal thickness was observed (Supplementary Fig. 3).

### Senescent fibroblasts induce hallmarks of skin aging in a 3D model

**Fig. 2:**
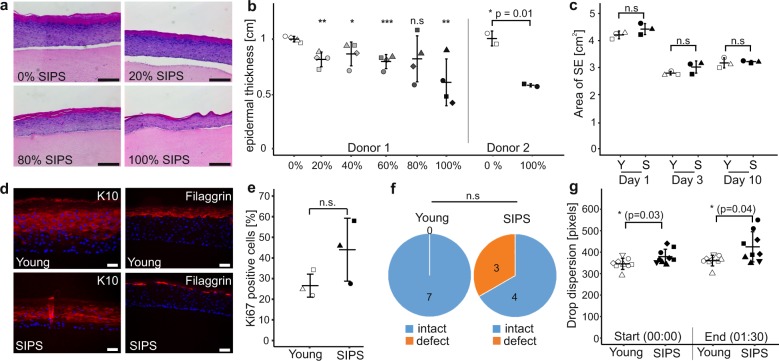
Senescent fibroblasts induce hallmarks of skin aging in a 3D model. **a**, **b** Reduction of epidermal thickness in senoskin equivalents. Senoskin equivalents were built by gradually replacing proliferating with senescent fibroblasts. **a** Representative H&E sections of Donor 1 are shown. Scale bar 200 µm. **b** For each sample, relative thickness of the whole skin-equivalent section (5–7 images each) was measured using ImageJ, and mean thickness was calculated. On the left side of the graph, four independent replicates were counted for each condition in a blinded fashion; on the right side, the experiment was repeated twice in a second fibroblast donor. Statistical analysis was performed for Donor 1 using ANOVA and Dunnett post hoc test against control (0% SIPS). For Donor 2, unpaired *t* test was used. Error bars indicate SD. n.s ≥ 0.05; *p < 0.05; **p < 0.01. **c** Contractibility of the dermis is not affected by the addition of senescent fibroblasts. SEs were built either with proliferating (Young) or senescent fibroblasts (SIPS). Pictures of the SEs were taken daily, and contraction of the dermis was evaluated by measuring the total area via ImageJ. Three independent replicates from one donor are shown. Statistical analysis was performed using unpaired *t* test; error bars indicate SD. n.s ≥ 0.05; *p < 0.05; **p < 0.01. **d** Evaluation of differentiation markers in senoskin equivalents. SEs were prepared according to the protocol and analyzed for the early differentiation marker Cytokeratin 10 (**d**, left) and late differentiation marker Filaggrin (**d**, right). Representative images of one experiment are shown. Scale bar 50 µm. **e** Evaluation of the proliferation marker Ki67 in senoskin equivalents. SEs were prepared according to the protocol, and Ki67-positive cells were counted in three independent replicates in a blinded fashion. Percentage versus total cells in the stratum basale are given. Statistical analysis was performed using unpaired *t* test; error bars indicate SD. n.s ≥ 0.05; *p < 0.05; **p < 0.01. **f** Integrity of barrier function. SEs were prepared according to the protocol and analyzed for barrier integrity by adding a drop of biotin on top of the sample for 1 h (37 °C, 7% CO_2_). The SEs were then embedded in paraffin, sectioned, and counterstained with a streptavidin-Alexa488 conjugate. Integrity of barrier function was evaluated in a blinded fashion, and converted into a yes/no statement. The results from seven independent replicates from one fibroblast donor are shown. Statistical analysis was performed using Fisher’s exact test, n.s ≥ 0.05. **g** Evaluation of surface properties using contact-angle measurement. Skin-equivalent biopsies (6 mm) were punched out and fixed on the contact- angle measurement stage. Via a syringe, 5 µl of water was added as a drop on top of the SE, and flow/leakage was recorded via camera for 1:30 min. Pictures at the start of the experiment (0:00 min) and at the end (1:30 min) were captured, and the area of the droplet was quantified via ImageJ. Nine independent replicates from one fibroblast donor are shown. Statistical analysis was performed using unpaired *t* test; error bars indicate SD. n.s ≥ 0.05; *p < 0.05; **p < 0.01.

In order to test for phenotypic changes caused by senescent fibroblasts, we next characterized the senoskin model regarding epidermal thickness, a hallmark of aged skin in vivo^10^. Indeed, the average epidermal thickness in hematoxylin- and eosin- (H&E) stained sections progressively decreased with increasing ratios of senescent cells (Fig. 2a, b), independent of oxidative stress-induced or doxorubicin-induced senescence (Supplementary Fig. 4). This decrease was not caused by a reduced contractibility of the underlying dermis (Fig. 2c). In all subsequent experiments, we further characterized only senoskins built with 100% senescent fibroblasts. This accommodates for the limited interaction time of 2 weeks between fibroblasts and keratinocytes in our senoskin model, which is considerable less time than months of coexistence in vivo.

To test our hypothesis that a reduction of the overall thickness might indicate a shift in the balance between proliferation and differentiation of cells within the senoskin, we performed immunohistochemistry stainings. Expression of markers for early (keratin 10) and late (filaggrin) differentiation was reduced in the presence of senescent fibroblasts (Fig. 2d). Moreover, areas of discontinuous filaggrin staining in the stratum corneum (Fig. 2d) were observed in senoskin models, suggesting modulation of terminal keratinocyte differentiation by senescent fibroblasts. On the other hand, there was a trend toward increased expression of the proliferation marker Ki67 in keratinocytes of senoskin (Fig. 2e).

Another hallmark of skin aging is loss of barrier function with increased age^11^. As defects in terminal differentiation of keratinocytes are likely to affect barrier integrity, we performed biotin permeability assays (Supplementary Fig. 5). While barrier function was intact in all SEs with early-passage fibroblasts, our senoskin model showed partial impairment (Fig. 2f). In accordance with this, literature on aged skin reports altered drug permeability and high interindividual variability^11^. As barrier function might be influenced by general surface properties, we adapted a contact-angle measurement method for SEs (Supplementary Fig. 6), which allows the visualization and quantification of liquid dispersion on surfaces. Indeed, there was a significant difference in drop dispersion when comparing senoskin versus control SEs. Furthermore, our senoskin model showed higher variability in drop dispersion at the end of the measurement (1:30 min, Fig. 2g). Taken together, these results indicate that senescent fibroblasts lead to different surface properties and reduced barrier integrity in skin equivalents, which might closely resemble the in vivo situation^11^.

Elasticity of the human skin is reduced with age, which results in tissue slackening and wrinkle formation^12^. We therefore investigated the micromechanical properties of single collagen fibers using indentation loading by atomic force microscopy^13^ (Supplementary Fig. 7); however, no significant difference in the elastic modulus between senoskin and the control group was observed.

### Distinct changes in the chemical composition of senoskin supernatants

**Fig. 3:**
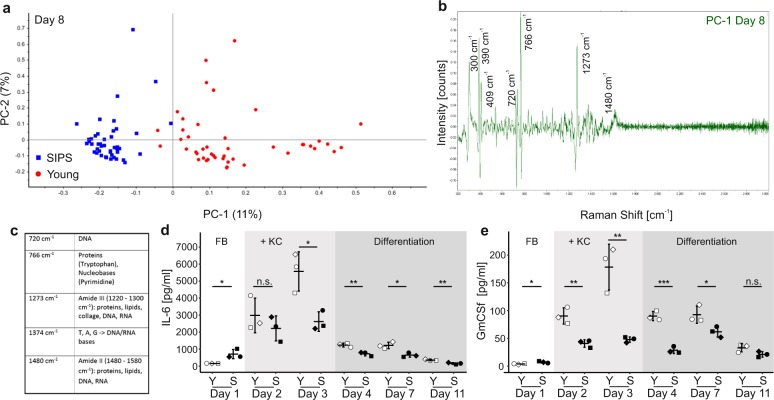
Distinct changes in the chemical composition of senoskin supernatants. **a–c** Raman microspectroscopy discriminates between supernatants from young and senoskin models. Raman spectra were enhanced using gold nanoparticles, which were dried overnight on a glass slide, before supernatants from young or senoskin equivalents (from day 8, differentiation phase) were added. Spectra were acquired with a 785-nm laser over a range of 400–3000 cm^−1^. A total of 15 spectra per sample were collected; three biological samples were measured per condition. The first derivative was calculated, and after unit vector normalization, spectra were subjected to principal component analysis (PCA, **a**). In **b**, the main differences in Raman spectra of supernatants from senoskins and young skin equivalents are visualized as peaks. Panel **c** gives an overview of the most prominent bands and their corresponding major components. **d**, **e** Cytokine pattern in supernatants of sensoskin equivalents. IL-6 (**d**) and GmCSF (**e**) levels were analyzed in supernatants from young and senoskin equivalents via ELISA. Samples were taken on days 1, 2, 3, 4, 7, and 11, whereas the first three time points reflect the growing phase of the experiment. Keratinocytes are added on day 2. On day 4, differentiation of KCs is induced via generation of an air–liquid interface. Per condition, three independent replicates from one donor are shown. Statistical analysis was performed using unpaired *t* test (Bonferroni corrected); error bars indicate SD. n.s ≥ 0.05; *p < 0.05; **p < 0.01.

Senescent cells are known to develop the senescence-associated secretory phenotype (SASP)^14^, which might mediate the effects of senescent fibroblasts on keratinocytes in our skin-equivalent model. To get an overview of the differences in the chemical composition of the secretome in senoskin models, Raman spectra of supernatants from young and senoskin equivalents were collected. PCA was clearly able to distinguish between spectra from supernatants from young and senoskin equivalents, indicating differences in the secretome (Fig. 3a). According to the loadings of the corresponding PCA (Fig. 3b, c), among others, amide II (1480–1580 cm^−1^) and amide III (1220–1300 cm^−1^) bands corresponding to proteins, lipids, and nucleic acids, as well as peaks between 600 and 900 cm^−1^ referring to nucleic acids, are responsible for the differences in the analyzed spectra. These findings are consistent with differences in Raman fingerprints at the cellular level between proliferating and senescent fibroblasts^15,16^. As additional control, we analyzed the supernatants of skin equivalents with low young fibroblast cell numbers, again to exclude that lower fibroblast density might confound the results. Principal component analysis (PCA) did not distinguish between spectra of supernatants originating from models with lower, compared with higher numbers of fibroblasts, indicating that the secretome of the models is not altered by different cell numbers (Supplementary Fig. 8).

We then proceeded to quantitate three examples of the SASP, such as interleukin-6 (IL-6), granulocyte macrophage colony-stimulating factor (GmCSF), and IL1-alpha in the supernatants of senoskin and control skin equivalents over time. As expected, IL-6 levels were fourfold increased in senoskin on the first day of culture (Fig. 3d), mimicking the increased levels in aged skin, which contribute to a pro-inflammatory state in vivo^14^. However, as soon as keratinocytes were added (day 3), a 35-fold increase in IL-6 levels in young skin equivalents was observed, while senoskin showed a blunted response of threefold only (Fig. 3d). During the differentiation phase from day 4–11, general reduction back to basal levels was observed. However, IL-6 levels remained twofold higher in young SEs compared with senoskin (day 11, Fig. 3d). The same trend was observed for GmCSF (Fig. 3e) as well as for IL-1alpha levels (Supplementary Fig. 9). Increased GmCSF and IL-6 levels are reported to be associated with induction of keratinocyte proliferation in wound healing^17,18^, a situation that is mimicked within this model system due to the formation of a “new” epidermal layer. The reduced inducibility of these factors in senoskin may be partially responsible for the reduced epidermal thickness we observed. A similar behavior of IL-6 levels (Supplementary Fig. 10) during the differentiation phase was observed in senoskin generated using doxorubicin-induced senescent fibroblasts, suggesting a general phenomenon of cellular senescence in skin equivalents. Since no studies have yet addressed the response of SASP factors in a time course of wound healing in the presence of senescent cells, we consider this finding of high interest for follow-up studies. It could be speculated that even if basal levels of some cytokines are higher, blunted response to stimuli might also lower the regenerative capacity as is observed in the elderly. However, it remains unclear why more Ki67-positive epidermal cells were observed in the presence of senescent fibroblasts, even if this is in line with the observation that senescent cell-derived EVs promote proliferation of MSCs^19^ or keratinocytes^20^. In any case, further investigation is needed to analyze the interplay between senescent fibroblasts and keratinocytes regarding differentiation and proliferation in senoskin.

### Summary

In summary, we here present a novel method of generating skin equivalents that contain senescent fibroblasts and thereby show hallmarks of aged skin. Our senoskin model can serve to mimic aged skin in vitro to characterize the negative impact of cellular senescence on keratinocyte proliferation and differentiation capacity in 3D organotypic cultures. Furthermore, our model allows the evaluation of strategies to counteract such effects^21^.

## Methods

### Cell culture

Primary keratinocytes were kindly provided by the group of Michael Mildner, Medical University Vienna. The cells were routinely cultured in keratinocyte basal medium 2 (KBM-2) supplemented with KGM-2 SingleQuot^©^ kit (LONZA, Basel, CH) at 37 °C, 7% CO_2_, and 95% humid air. In addition, primary human dermal fibroblasts (HDF, Evercyte, GmbH) were cultured in DMEM/HAMs (1:1, Biochrom, Berlin, GER) supplemented with 10% fetal calf serum (FCS) and 4 mM L-glutamine at 37 °C, 7% CO_2_, and 95% humid air.

All cells were split at a ratio of 1:3–1:4 twice a week and periodically tested for the absence of mycoplasma. The growth of cells was monitored routinely by cell counting (ViCell X-R, Beckman-Coulter, CA, USA) at each passaging. Population doublings were calculated using the formula *n* = 3.32 × (log (cell count) − log (seeded cells)), whereas *n* is the final population-doubling number^22^.

### SIPS

Cells were seeded at a cell density of 3500 cells/cm^2^ one day prior to the treatment. For senescence induction with H_2_O_2_, the cells were treated nine times with 100 µM H_2_O_2_ supplemented to the media for 1 h followed by a media change. For senescence induction with doxorubicin, cells were treated two times with 200 nM doxorubicin. Recovery of cells after treatment was done for 2 weeks with media change once a week, before cells were used for experiments. Induction of premature senescence was verified by SA-β-gal staining, p21 expression, and the absence of BrdU incorporation. SIPS HDFs were cultured and monitored for over 50 days to assure that the induced growth arrest was permanent.

### SA-β-gal staining

SA-β-gal staining was performed according to standard procedures^2^. Fifteen random images were taken per well at ×100 magnification, and after randomization, positive and negative cells were counted in a blinded fashion.

### RNA isolation and RT-qPCR

Cells were lysed in TRI Reagent (Sigma, St. Louis, MO, USA), and RNA was isolated following the manufacturer’s protocol. RNA concentration and quality were measured with a ND-1000 NanoDrop spectrometer. cDNA was synthesized from 500 ng of total RNA using High-Capacity cDNA Reverse Transcription Kit (ThermoFisher, MA, USA) and quantified with 5x HOT FIREPol^®^ EvaGreen^®^ qPCR Mix Plus with ROX (Solis BioDyne, Tartu, Estonia) using a Rotor-Gene Q cycler (Qiagen, Hilden, Germany). Expression values were normalized to GAPDH mRNA.

### BrdU staining

Cells were seeded two days prior to the experiment and treated on the following day with 10 µM BrdU for 24 h. Then, cells were harvested, and 300,000 cells were fixed with ice-cold 70% ethanol. Cells were stored overnight at 4 °C, then permeabilized with 2 M HCl/1% Triton X-100 for 30 min. Afterwards, the cell suspension was neutralized with 0.1 M Na-borate (pH 8.5), stained for 30 min with monoclonal mouse anti-BrdU antibody (Becton Dickinson, CA, USA), and counterstained with an anti-mouse FITC-conjugated antibody (1:100, Sigma-Aldrich, MO, USA). For analysis of cell cycle distribution, cells were counterstained with 2.5 µg of propidium iodide (PI)/ml and analyzed by flow cytometry using a Gallios Flow Cytometer (Beckman-Coulter). Quantification of DNA histograms was done using KALUZA software (Becton Dickinson, CA, USA).

### Skin equivalents

Skin equivalents were prepared as published by Mildner et al.^7^. Briefly, either 2.5 × 10^5^ young (pD < 16, control) or senescent HDF (senoskin) were seeded in a collagen gel consisting of eight parts of collagen G (Biochrome, Berlin, DE), one part of 10× HBSS (ThermoFisher Scientific, MA, USA), and one part of FCS (Sigma-Aldrich, MO, USA). The gel was equilibrated overnight with KGM-2 supplemented with KGM-2 Bullet Kit (Lonza, Basel, CH) followed by a keratinocyte overlay of 1.5 × 10^6^ cells on day 2. The so-formed early skin equivalents were then lifted to the air–liquid interface to start differentiation on day 3. The differentiation media (KGM, Lonza, Basel, CH) was supplemented with all components of the KGM BulletKit (Lonza, Basel, CH), except for bovine pituitary extract. In addition, 1.15 mM CaCl_2_, 50 µg/ml L-ascorbic acid, 0.1% bovine serum albumin, and 10 µg/ml transferrin (all Sigma, St. Louis, MO, USA) were added. The differentiation medium was refreshed every other day throughout the whole differentiation process (day 3–10), and samples were taken daily for multiplex ELISA. After 10 days, the skin equivalents were harvested, formalin-fixed, and paraffin-embedded for further histological analysis. All skin-equivalent experiments were performed in duplicates and were repeated three times.

For re-isolation of fibroblasts from skin equivalents, the epidermis was removed from the dermal, fibroblast-containing part. Then, collagen was digested for 30–60 min at 37 °C with Collagenase IV (Sigma, St. Louis, MO, USA). Cells were pelleted (1000 rpm, 5 min), and liquid collagen was removed. Cells were resuspended in 200 µl media, manually counted, and reseeded in six-well plates to analyze reattachment.

### Skin equivalents—area calculation

For evaluation of skin-equivalent area, pictures were taken daily as before. For reference and to ensure consistent size of images, a ruler was placed next to the cell culture plate. The total area was calculated using ImageJ in a blinded fashion.

### Immunhistochemistry

All stainings were performed on 5-µm-thick sections of formalin-fixed, paraffin-embedded tissues. A standard hematoxylin and eosin (H&E) staining technique was used for histological analysis. For immunohistochemistry analysis, paraffin was removed, samples rehydrated, and blocked of endogenous peroxidase with 0.3% hydrogen peroxide. Antigen retrieval with 10 mM citrate buffer (containing 0.05% Tween 20, pH 6.0) was performed for 30 min at 80 °C. The sections were then incubated with the first antibody in PBS/2% BSA overnight at 4 °C. After washing with PBS, slides were incubated with the corresponding secondary antibodies in PBS/2% BSA for 1 h at room temperature. The slides for fluorescence analysis were counterstained with DAPI (1:5000, ThermoFisher Scientific, MA, USA), mounted with Fluoprep (bioMérieux, Marcy l’Etoile, France), and analyzed by fluorescence microscopy on a Leica DMI-6000 microscope. Alternatively, visualization was done by treating slides with HRP-conjugated secondary antibodies with ultravision-labeled horseradish peroxidase (HRP) polymer (UVLP, Dako, Glostrup, Denmark) for 15 min. Then, antibody binding was visualized with DAB+ chromogen and counterstained with hematoxylin.

### Antibodies used for immunofluorescence and IHC

The following antibodies were used: Ki67 (mouse, DAKO M720, 1:100), Filaggrin (mouse, LEICA NLC-FIL, 1:100), Cytokeratin 10 (rabbit, Biolegends 905401, 1:1000), and Alexa Fluor 488 anti-mouse igG (H + L) (donkey, Jackson Immunoresearch 715-545-150, 1:500).

### Barrier function assay

To evaluate barrier function, a small section from the middle of the fully formed skin equivalents was punched out (6 mm). A drop of biotin (15 µl) was placed on top, and samples were incubated at 37 °C, 5% CO_2_ for 1 h. Then, the remaining biotin was removed; samples were processed, embedded, and cut. For staining, paraffin was removed, and samples rehydrated as stated above. Penetration of biotin into the tissue was visualized using a Streptavidin-Alexa488 conjugate (LifeTech, Carlsbad, CA, USA).

### Drop-dispersion assay (contact-angle measurement)

Skin-equivalent biopsies (6 mm) were punched out and fixed on the contact-angle measurement stage. Via a syringe, 5 µl of water was added as a drop on top of the SE, and flow/leakage was recorded via camera for 1:30 min. Pictures at the start of the experiment (0:00 min) and at the end (1:30 min) were captured, and the area of the droplet was quantified via ImageJ.

### Cytokine measurement

Human cytokines (IL-8, IL-6, and GM-CSF as indicated) were measured by Luminex using specific matched-pair antibodies and recombinant cytokines as standards (Merck Millipore, Billerica, MA).

### Raman microspectroscopy

Fifteen microliters of gold nanoparticles (Gold, 40-nm 9.00 × 10^10^ particles per ml, BBI Solutions) were dried overnight in a µ-Slide with glass bottom. The supernatant from skin equivalents, stored at −80 °C, was added freshly prior to measurements. Spectra acquisition was conducted using an XploRA INV Raman microscope (Horiba Jobin Yvon, Benshein, Germany) with a 785-nm laser, 1200 gr/mm grating, and CFI Plan APOx100 NA 1.4 Oil objective (Nikon). The laser beam was focused on close proximity to an aggregate of gold particles. Per pixel, two spectra over the range of 400–3000 cm^–1^ were acquired with an integration time of 10 s. A total of 15 spectra per sample were collected. The first derivative was calculated, and after unit vector normalization, spectra were used for principal component analysis (PCA). LabSpec 6 (Horiba) was used for spectra acquisition. The Unscrambler^®^ X (CAMO Software) was used for processing and analysis of spectra.

### Atomic force microscopy cantilever-based microindentation

The indentation modulus of fully hydrated samples (in the form of tissue cryosections deposited on glass slides) was subsequently assessed with atomic force microscopy (AFM) cantilever-based microindentation experiments by employing a NanoWizard SpeedA (JPK Instruments-Bruker, Berlin) AFM instrument. For microindentation tests, a colloidal probe (borosilicate glass) of 11.4 μm in diameter (*D*_sphere_) was attached onto a cantilever of 0.0298 N/m spring constant. Quasi-static load–unload ramp was performed, and the resulting force-indentation curves were analyzed similarly to a previous published work^13^ by using the Oliver–Pharr method^23^. Briefly, the unloading stiffness (S) was determined from the upper part of the unloading slope of the force-indentation curve. The contact depth (*h*_c_) was also calculated based on the Oliver–Pharr method, and given the spherical shape of the colloidal probe, the projected area of contact (*A*_c_ = *π*(*D*_sphere_ *h*_c_ – *h*_c_^2^)) was determined. With these measurements, the indentation modulus of the sample (*E*_sample_) is determined through the equation *E*_sample_ = (sqrt(*π*)/(2β))*(1 − v^2^)*S/sqrt(*A*_c_).

### Reporting summary

Further information on research design is available in the Nature Research Reporting Summary linked to this article.

## Supplementary information

Supplementary Figures

Reporting Summary

## Data Availability

The datasets generated and/or analyzed during this study are available from the corresponding author on reasonable request.
