# Supplementary material for: Organotypic human skin culture models constructed with senescent fibroblasts show hallmarks of skin aging
Source: NPJ Aging Mech Dis. 2020 Mar 6;6:4. doi: 10.1038/s41514-020-0042-x (PMC7060247; doi:10.1038/s41514-020-0042-x)
Supplement: Supplementary file 1 — Supplementary Figures [file 41514_2020_42_MOESM1_ESM.pdf]

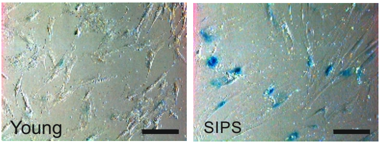

Supplementary Figure 1

Quantification of senescence associated beta-galactosidase (SA-β-Gal). Human dermal fibroblasts (HDF) were seeded at a density of 3.500 cells/cm^2 and stressed twice for five consecutive days with 100 µM H_2O_2 each (SIPS). After recovery for 3 days, cells were stained for SA-β-Gal. One representative image per condition is shown.

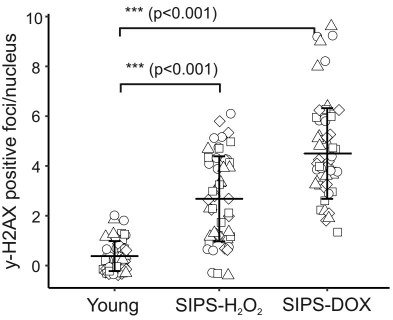

Supplementary Figure 2

Quantification of γ-H2AX positive foci in senescent cells. Human dermal fibroblasts were stressed either with H_2O_2 or doxorubicin according to protocol. Cells were stained for γ-H2AX and foci in the nucleus of fifty cells per condition were counted in a blinded fashion. Statistical analysis was performed using unpaired t-test (bonferroni corrected), error bars indicate SD. n.s ≥ 0.05; *p < 0.05; **p < 0.01. Data from one representative experiment is shown. The experiment was repeated three times each in 3 different donors with similar results.

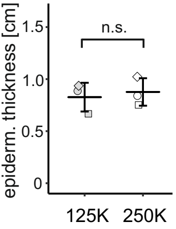

Supplementary Figure 3

Evaluation of epidermal thickness in skin equivalents built with a reduced amount of fibroblasts. Skin equivalents were either built with 250.000 fibroblasts/equivalent (control, 250K) or 125.000 fibroblasts/equivalents (125K) in biological triplicates. For each sample, relative thickness of the whole skin equivalent section (5-7 images each) was measured using ImageJ in a blinded fashion and mean thickness was calculated. Statistical analysis was performed using unpaired t-test. Error bars indicate SD. n.s ≥ 0.05; *$p < 0.05$; **$p < 0.01$.

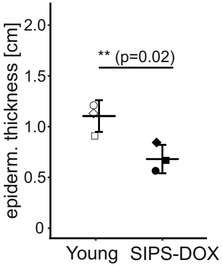

Supplementary Figure 4

Doxorubicin-induced senescent fibroblasts reduce epidermal thickness in the senoskin model. Senoskin equivalents were built by adding Doxorubicin-induced senescent fibroblasts into the dermal matrix. After seven days of differentiation, skin equivalents were harvested, paraffin embedded, sectioned and stained with H&E. For each senoskin, relative thickness of the whole skin equivalent section (5-7 images each) was measured using ImageJ and mean thickness was calculated. Three independent replicates were counted for each condition in a blinded fashion. Statistical analysis was performed using unpaired t-test. Error bars indicate SD. n.s ≥ 0.05; *$p < 0.05$; **$p < 0.01$.

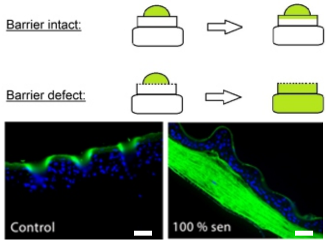

<u>**Supplementary Figure 5**</u>

Schematic depiction of a barrier assay. SE were prepared according to protocol and analyzed for barrier integrity by adding a drop of biotin on top of the sample for one hour (37°C, 7% CO_2). The SE were then embedded in paraffin, sectioned and counterstained with a streptavidin-Alexa Fluor 488 conjugate. If the barrier is defect, the whole skin equivalent will be stained (SIPS), otherwise only the stratum corneum will stain positive for biotin (Young).

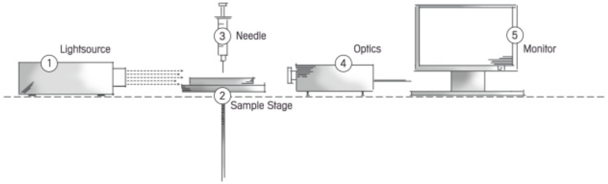

<u>**Supplementary Figure 6**</u>

Schematic depiction of contact angle measurement setup.

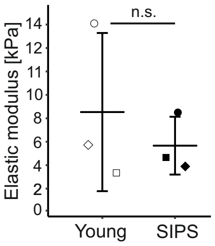

Supplementary Figure 7

Atomic force microcopy cantilever-based microindentation. The indentation modulus of fully hydrated samples (in the form of tissue cryosections deposited on glass slides) was assessed with atomic force microscopy (AFM) cantilever-based microindentation experiments. For microindentation tests, a colloidal probe (borosilicate glass) was attached onto a cantilever. Quasi-static load-unload ramp was performed and the resulting force-indentation curves were analyzed similarly to a previous published work and using the Oliver-Pharr method. Per sample, four different spots were measured and mean indentation modulus was calculated. Three independent replicates from one donor are shown. Statistical analysis was performed using unpaired t-test, error bars indicate SD. n.s ≥ 0.05; *$p < 0.05$; **$p < 0.01$.

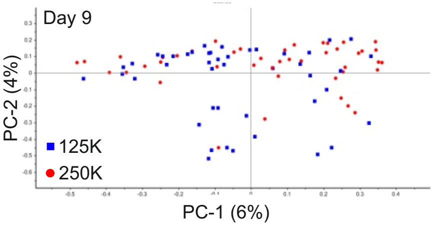

<u>Supplementary Figure 8</u>

Raman microspectroscopy analysis of supernatants from skin equivalents built with a reduced amount of fibroblasts. Skin equivalents were either built with 250.000 fibroblasts/equivalent (control, 250K) or 125.000 fibroblasts/equivalents (125K) in biological triplicates. Supernatants from day 9 (differentiation phase) were added onto gold particles that were dried overnight. Spectra were acquired with a 785nm laser over a range of 400 – 3000 cm^{-1}. A total of 15 spectra per sample was collected. The 1st derivative was calculated and after unit vector normalization, spectra were subjected to principle component analysis (PCA).

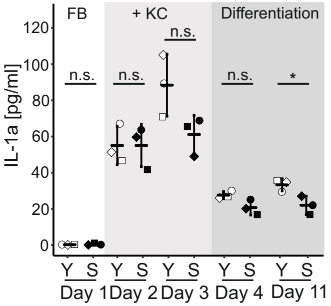

Supplementary Figure 9

Cytokine pattern in supernatants of senoskin equivalents. IL-1a levels were analyzed in supernatants from young and senoskin equivalents via ELISA. Samples were taken on day 1, 2, 3, 4, 7, 11 whereas the first 3 time points reflect the growing phase of the experiment while the differentiation of the cells was started on day 4. Per condition, three independent replicates are shown. Statistical analysis was performed using unpaired t-test (bonferroni corrected), error bars indicate SD. n.s ≥ 0.05; *$p < 0.05$; **$p < 0.01$.

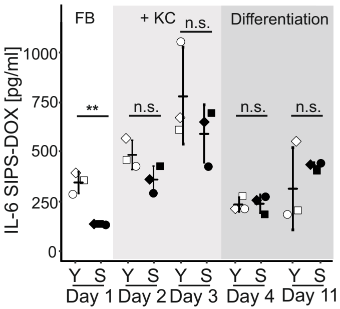

Supplementary Figure 10

Cytokine pattern in supernatants of doxorubicin-induced senoskin equivalents. IL-6 levels were analyzed in supernatants from young and doxorubicin-induced senoskin equivalents via ELISA. Samples were taken on day 1, 2, 3, 4, 7, 11 whereas the first 3 time points reflect the growing phase of the experiment while the differentiation of the cells was started on day 4. Per condition, three independent replicates are shown. Statistical analysis was performed using unpaired t-test (bonferroni corrected), error bars indicate SD. n.s ≥ 0.05; *$p < 0.05$; **$p < 0.01$.
